# Investigating the Use of Pretrained Convolutional Neural Network on Cross-Subject and Cross-Dataset EEG Emotion Recognition

**DOI:** 10.3390/s20072034

**Published:** 2020-04-04

**Authors:** Yucel Cimtay, Erhan Ekmekcioglu

**Affiliations:** Institute for Digital Technologies, Loughborough University London, London E20 3BS, UK; e.ekmekcioglu@lboro.ac.uk

**Keywords:** EEG, emotion recognition, pretrained models, convolutional neural network, dense layer, subject independency, dataset independency, raw data, filtering on output

## Abstract

The electroencephalogram (EEG) has great attraction in emotion recognition studies due to its resistance to deceptive actions of humans. This is one of the most significant advantages of brain signals in comparison to visual or speech signals in the emotion recognition context. A major challenge in EEG-based emotion recognition is that EEG recordings exhibit varying distributions for different people as well as for the same person at different time instances. This nonstationary nature of EEG limits the accuracy of it when subject independency is the priority. The aim of this study is to increase the subject-independent recognition accuracy by exploiting pretrained state-of-the-art Convolutional Neural Network (CNN) architectures. Unlike similar studies that extract spectral band power features from the EEG readings, raw EEG data is used in our study after applying windowing, pre-adjustments and normalization. Removing manual feature extraction from the training system overcomes the risk of eliminating hidden features in the raw data and helps leverage the deep neural network’s power in uncovering unknown features. To improve the classification accuracy further, a median filter is used to eliminate the false detections along a prediction interval of emotions. This method yields a mean cross-subject accuracy of 86.56% and 78.34% on the Shanghai Jiao Tong University Emotion EEG Dataset (SEED) for two and three emotion classes, respectively. It also yields a mean cross-subject accuracy of 72.81% on the Database for Emotion Analysis using Physiological Signals (DEAP) and 81.8% on the Loughborough University Multimodal Emotion Dataset (LUMED) for two emotion classes. Furthermore, the recognition model that has been trained using the SEED dataset was tested with the DEAP dataset, which yields a mean prediction accuracy of 58.1% across all subjects and emotion classes. Results show that in terms of classification accuracy, the proposed approach is superior to, or on par with, the reference subject-independent EEG emotion recognition studies identified in literature and has limited complexity due to the elimination of the need for feature extraction.

## 1. Introduction

The electroencephalogram (EEG) is the measurement of the electrical signals which are a result of brain activities. The voltage difference is measured between the actual electrode and reference electrode. There are several EEG measurement devices in the market such as Neurosky, Emotiv, Neuroelectrics and Biosemi [1] which provide different spatial and temporal resolutions. Spatial resolution is related to number of electrodes and temporal resolution is related to the number of EEG samples processed for unit time. Generally, EEG has high temporal but low spatial resolution. In terms of spatial resolution, EEG electrodes can be placed on the skull according to the 10-20 or 10-10 and 10-5 positioning standards [2].

EEG has lately been used as a powerful emotion prediction modality. It is reliable, portable and relatively inexpensive compared to other brain monitoring tools and technologies. EEG has many application areas. For the clinical applications, EEG is mostly used to investigate the patterns related to sleep [3] and epilepsy [4]. Some other applications of EEG analysis are consciousness and hyperactivity disorders [5,6], measurement of the affective components such as level of attention [7,8,9], mental workload [10], mood and emotions [11,12,13,14] and brain computer interfaces which involve the work of transforming brain signals into direct instructions [15,16,17].

Human emotions have crucial effects on communication with others. Understanding the emotions of humans provides a way to control and regulate behaviors. The name of emotional recognition in the digital world is affective computing, which is the technique of emotion recognition using various sensors and computer-based environments. This concept was originated with Rosalind Picard’s paper [18] on affective computing in 1995. In the EEG context, affective computing is achieved by setting up brain computer interfaces (BCI) which includes sensors, machines and coding. In BCI, the operation of affective computing starts with presenting users with stimuli which induces specific emotions. These stimuli may be video, image, music, etc. During the session, EEG data are recorded with EEG devices. The next step is typically extracting features from the recorded EEG and training a classifier to predict emotion labels. The final step is testing the trained model with new EEG data which are not used in training session. Data collection and labelling are the most important aspects which has an impact on resulting recognition accuracy. The “Brouwer recommendations” about data collection given in [19] are crucial for handling accurate data and labelling.

In relevant emotion recognition literature, emotions have been broadly represented in two ways. The first approach classifies emotions as discrete states such as the six basic emotions proposed by Ekman and Friesen [20]. The second approach defines emotion as a continuous 4-D space of valence, arousal, dominance and liking [21,22]. In most of the studies, this space is reduced to 2-D as valence and arousal dimensions [23,24]. The study conducted in [25] is very useful in the sense that it relates discrete and continuous approaches to each other. Discrete emotion states are mapped on to the valence-arousal circumplex model according to high number of blogposts. This study enables scientists to transform emotions from continuous space to discrete space.

In EEG data channels, typical frequency domain analysis is used. In the frequency domain, the most important frequency bands are delta (1–3 Hz), theta (4–7 Hz), alpha (8–13 Hz), beta (14–30 Hz) and gamma (31–50 Hz) [26]. Fast Fourier Transform (FFT), Wavelet Transform (WT), eigenvector and autoregressive are the methods which transform EEG signal from time domain to frequency domain [27]. The study [28] extracts several frequency–domain features like Differential Entropy (DE) and Energy Spectrum (ES) in order to classify EEG data and [29] investigate the critical frequency bands and channels for EEG-based emotion recognition. One of the major problems we observed in the context of emotion classification based on the analysis of EEG channels was that the classifier performance fluctuates remarkably across persons as well as across dataset. Most of these approaches train their classifiers using a set of features derived using the frequency domain analysis. While the classifiers’ performances are sufficiently high on test data, which comprise samples belonging to the same subjects but excluded from training and validation, when the same classifier is applied on EEG data of other subjects or on data extracted from various other datasets, the performance is degraded significantly. The same problem in subject-independent analysis is not apparent in literature in the context of emotion recognition from facial expressions or other physiological data (e.g., heart rate variability, electro-dermal activity). This observation has led us to investigate classification approaches using raw EEG data, which preserves all information and prevents the risk of removing hidden features before training the classifier.

## 2. Literature Review

For the classification of EEG signals, many machine learning methods such as K-Nearest Neighbor (KNN) [28], Support Vector Machine (SVM) [28,29,30], Decision Tree (DT) [31], Random Forest (RF) [32] and Linear Discriminant Analysis (LDA) [33] are applied. In the deep learning context, DBN (Deep Belief Network) [34] and AE (Auto Encoders) [35] are studied with promising results. Besides DBN and AE, Convolutional Neural Network (CNN) and Long-Short Term Memory (LSTM) structures are widely used [36,37,38,39,40]. Most of these models have shown good results for subject dependent analysis. In [41], KNN method is employed on the DEAP dataset [42] for different numbers of channels which show accuracy between 82% and 88%. The study conducted in [43] quadratic time-frequency distribution (QTFD) is employed to handle a high-resolution time-frequency representation of the EEG and the spectral variations over time. It reports mean classification accuracies ranging between 73.8% and 86.2%. In [44], four different emotional states (happy, sad, angry and relaxed) are classified. In that study, Discrete Wavelet Transform (DWT) is applied on the DEAP dataset. Wavelet features are classified using a Support Vector Machine (SVM) classifier with Particle Swarm Optimization (PSO) [45]. The overall accuracy of 80.63% is reported with valence and arousal accuracy of 86.25% and 88.125%, respectively.

An important issue in EEG-based emotion detection is the non-linearity and non-stationarity EEG signals. Feature sets, such as the spectral band powers of EEG channels, extracted from different people against the same emotional states do not exhibit strong correlations. For example, galvanic skin response is a robust indicator of arousal state, where different people’s responses correlate with each other well. Training and testing data made of EEG channels’ spectral band powers and their derivatives have different distributions. And it is difficult to identify sets of features from the EEG recordings of different subjects, different sessions and different datasets that exhibit more commonality. This makes the classification difficult with traditional classification methods, which assume identical distribution. In order to address this problem and provide subject independency to EEG-based emotion recognition models, deeper networks, domain adaptation and hybrid methods have been applied [46,47]. Furthermore, various feature extraction techniques have been applied, and different feature combinations have been tried [48].

Subject-independent EEG emotion recognition, as a challenging task, has gained high interest by many researchers recently. The method called Transfer Component Analysis (TCA) conducted in [46] reproduces Kernel Hilbert Space, on the assumption that there exists a feature mapping between source and target domain. A Subspace Alignment Auto-Encoder (SAAE), which uses non-linear transformation and consistency constraint method, is used in [47]. This study compares the results with TCA. It achieves a leave-one-out mean accuracy of 77.88% in comparison with TCA, which shows 73.82% on the SEED dataset. Moreover, mean classification accuracy for session-to-session evaluation is 81.81%, an improvement of up to 1.62% compared to the best baseline TCA. In one of the studies, CNN with the deep domain confusion technique is applied on the SEED dataset [49] and achieves 90.59% and 82.16 mean accuracy for conventional (subject-dependent) EEG emotion recognition and “leave one out cross validation”, respectively [50]. Variational Mode Decomposition (VMD) [51] is used as a feature extraction technique and Deep Neural Network as the classifier. It gives 61.25% and 62.50% accuracy on the DEAP dataset for arousal and valence, respectively. Another study, [52], uses a deep convolutional neural network with changing numbers of convolutional layers on raw EEG data which are collected during music listening. It reports maximum 10-fold-validation mean accuracy of 81.54% and 86.87% for arousal and valence, respectively. It also achieves 56.22% of arousal and 68.75% of valence accuracies for one-subject-out test. As can be seen, the reported mean accuracy levels drop considerable in one-subject-out tests due to the nature of the EEG signals.

The study [48] extracts totally 10 different linear and nonlinear features from EEG signals. The linear features are Hjorth activity, Hjorth mobility, Hjorth complexity, the standard deviation, PSD-Alpha, PSD Beta, PSD-Gamma, PSD-Theta, and the nonlinear features are sample entropy and wavelet entropy. By using a method called Significance Test/Sequential Backward Selection and the Support Vector Machine (ST-SBSSVM), which is a combination of the significance test, sequential backward selection, and the support vector machine, it achieves 72% cross subject accuracy for the DEAP dataset with high–low valence classification. It also achieves 89% maximum cross subject accuracy for the SEED dataset with positive–negative emotions. Another study [53] uses FAWT (Flexible Analytic Wavelet Transform) which decomposes EEG signals into sub bands. Random forest and SVM are used for classification. The mean classification accuracies are 90.48% for positive/neutral/negative (three classes) in the SEED dataset; 79.95% for high arousal (HA)/low arousal (LA) (two classes); 79.99% for the high valence (HV)/low valence (LV) (two classes); and 71.43% for HVHA/HVLA/LVLA/LVHA (four classes) in the DEAP dataset. In [54], the transfer recursive feature elimination (T-RFE) technique is used to determine a set of the most robust EEG features for stable distribution across subjects. This method is validated on DEAP dataset, the classification accuracy and F-score for arousal is 0.7867, 0.7526 and 0.7875, 0.8077 for valence. A regularized graph neural network (RGNN) is applied in [55] for EEG-based emotion recognition, which includes inter-channel relations. The classification accuracy results on the SEED dataset are 64.88%, 60.69%, 60.84%, 74.96%, 77.50%, 85.30% for delta, theta, alpha, beta, gamma and all bands. Moreover, it achieves 73.84% of accuracy on the SEED [49] dataset.

There are several studies which apply transfer learning, which aims to explore common stable features and apply to other subjects [56]. In terms of affective computing, the work is exploring some common and stable features which are invariant between subjects. This is also called domain adaptation. In [57], the scientists tried to find typical spatial pattern filters from various recording sessions and applied these filters on the following ongoing EEG samples. Subject-dependent spatial and temporal filters were derived from 45 subjects and a representative subset is chosen in [58]. The study [59] uses compound common spatial patterns which are the sum of covariance matrices. The aim of this technique is to utilize the common information which is shared between different subjects. The other important studies which apply different domain adaptation techniques on the SEED dataset are [47,60,61,62]. The common properties of these domain adaptation techniques are the exploration of an invariant feature subspace which reduces the inconsistencies of EEG data between subjects or different sessions. In the study in [63], the domain adaptation technique is applied not only in cross-subject context but also for cross datasets. The trained model in SEED dataset is tested against the DEAP dataset and vice versa. It reports an accuracy improvement of 7.25–13.40% with domain adaptation compared to the one without domain adaptation. Scientists applied an adaptive subspace feature matching (ASFM) in [62] in order to integrate both the marginal and conditional distributions within a unified framework. This method achieves 83.51%, 76.68% and 81.20% classification accuracies for the first, second and third sessions of the SEED dataset, respectively. This study also conducts testing between sessions. For instance, it trains the model with the data of first session and test on the second session data. In the domain adaptation method, the conversion of features into a common subspace may lead to data loss. In order to avoid this, a Deep Domain Confusion (DDC) method based on CNN architecture is used [64]. This study uses adaptive layer and domain confusion loss based on Maximum Mean Discrepancy (MMD) to automatically learn a representation, jointly trained to optimize classification and domain invariance. The advantage of this is adaptive classification, retaining the original distribution information.

Having observed that the distribution of commonly derived sets of features from EEG signals show differences between subjects, sessions and datasets, we anticipate that there could be some invariant feature sets that follow common trajectories across subjects, sessions and datasets. There is a lack of studies that investigate these additional feature sets in the EEG signals that can contribute to robust emotion recognition across subjects. The aim of this study is to uncover these kinds of features in order to achieve promising cross-subject EEG-based emotion classification accuracy with manageable processing loads. For this purpose, raw EEG channel recordings after normalization and a state-of-the-art pretrained CNN model are deployed. The main motivation behind choosing a pretrained CNN architecture is due to their superiority in feature extraction and inherent exploitation of the domain adaptation. To improve the emotion recognition accuracy, additionally in the test phase, a median filter is used in order to reduce the evident false alarms.

### Contributions of the Work

The contributions of this work to related literature in EEG-based emotion recognition can be summarized as follows:

- Feature extraction process is completely left to a pretrained state-of-the-art CNN model InceptionResnetV2 whose capability of feature extraction is shown as highly competent in various classification tasks. This enables the model to explore useful and hidden features for classification.
- Data normalization is applied in order to remove the effects of fluctuations in the voltage amplitude and protect the proposed network against probable ill-conditioned situations.
- Extra pooling and dense layers are added to the pretrained CNN model in order to increase its depth, so that the classification capability is enhanced.
- The output of the network is post-filtered in order to remove the false alarms, which may emerge in short intervals of time where the emotions are assumed to remain mostly unchanged.

## 3. Materials

The EEG datasets used in this work are SEED [49], the EEG data of DEAP [42] and our own EEG dataset [65] which is a part of the multimodal emotional database LUMED (Loughborough University Multimodal Emotion Database). All the datasets are open to public access.

### 3.1. Overview of the SEED Dataset

SEED dataset is a collection of EEG recordings prepared by the Brain-like Computing & Machine Intelligence (BCMI) laboratory of Shanghai Jiao Tong University. A total of 15 clips are chosen for eliciting (neutral, negative and positive) emotions. Each stimuli session is composed of 5 s of hint of movie, 4 min of clip, 45 s of self-assessment and 15 s of rest. There are 15 Chinese subjects (7 females and 8 males) participated in this study. Each participant had 3 sessions on different days. In total, 45 sessions of EEG data was recorded. The labels are given according to the clip contents (−1 for negative, 0 for neutral and 1 for positive). The data were collected via 62 channels which are placed according to 10–20 system, down-sampled to 200 Hz, a bandpass frequency filter from 0–75 Hz was applied and presented as MATLAB “mat” files.

### 3.2. Overview of the DEAP Dataset

DEAP [22] is a multimodal dataset which includes the electroencephalogram and peripheral physiological signals of 32 participants. For 22 of the 32 participants, frontal face video was also recorded. Data were recorded while minute-long music videos were watched by participants. A total of 40 videos were shown to each participant. The videos were rated by the participants in terms of levels of arousal, valence, like/dislike, dominance and familiarity which changes between 1 and 9. EEG data are collected with 32 electrodes. The data were down-sampled to 128 Hz, EOG artefacts were removed, a bandpass frequency filter from 4.0–45.0 Hz was applied. The data were segmented into 60 s intervals and a 3 s baseline data were removed.

### 3.3. Overview of the LUMED Dataset

The LUMED (Loughborough University Multimodal Emotion Dataset) is a new multimodal dataset that was created in Loughborough University, London (UK), by collecting simultaneous multimodal data from 11 participants (4 females and 7 males). The modalities include visual data (face RGB), peripheral physiological signals (galvanic skin response, heartbeat, temperature) and EEG. These data were collected from participants while they were presented with audio–visual stimuli loaded with different emotional content. Each data collection session lasted approximately 16 min long that consists of short video clips playing one after the other. The longest clip was approximately 2.5 min and the shortest one was 1 min long. Between each clip, in order to provide the participant a refresh and rest, a 20 s-long gray screen was displayed. Although the emotional ground truth of each clip was estimated based on the content, in reality, a range of different emotions might be triggered for different participants. For this purpose, after each session, the participants were asked to label the clips they watched with the most dominant emotional state they felt. In this current study, we exploited the EEG modality of the LUMED dataset only. For this study, we have re-labelled the samples, such that only 2 classes were defined as negative valence and positive valence. This is done to make a fair comparison with other studies. Moreover, each channel’s data were filtered in the frequency range of 0.5 Hz to 75 Hz to attenuate the high frequency components that are not believed to be have a meaningful correlation with the emotion classes. Normally, captured EEG signals are noisy with EMG (electromyogram) and EOG (electrooculogram) type artefacts. EMG artefacts are electrical noises resulting from facial muscle activities and EOG is electrical noise due to eye movements. For traditional classification and data analysis methods, in order to prevent heavily skewed results, these kinds of artefacts should be removed from the EEG channel data through several filtering stages. As an example, the study in [66] removes the eye movement artefacts from signal by applying ICA (Independent Component Analysis). The LUMED dataset was created initially with the purpose of training a deep-learning based emotion recognition system, described in Section 4. Depending on the type and purpose of other supervised machine learning systems, this dataset could require a more thorough pre-processing for artefact removal. In LUMED, EEG data was captured based on 10–20 system by Neuroelectrics Enobio 8 [67], an 8-channel EEG device with a temporal resolution of 500 Hz. The used channels were FP1, AF4, FZ, T7, C4, T8, P3, OZ, which are spread over frontal, temporal and center lobes of the brain.

## 4. Proposed Method

In this work, the emotion recognition model works on raw EEG signals without pre-feature extraction. Feature extraction is left to a state-of-the-art CNN model: InceptionResnetV2. The success of this pretrained CNN model on raw data classification was extensively outlined in [68]. Since the distribution of EEG data shows variations from person to person, session to session and dataset to dataset, it is difficult to identify a feature set that exhibits good accuracy every time. On the other hand, pretrained CNN models are very competent in feature extraction. Therefore, this work makes use of it.

### 4.1. Windowing of Data

**Figure 1:**
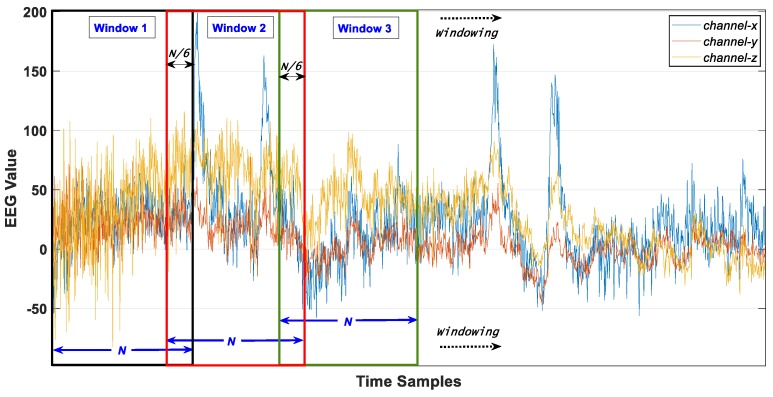
Windowing with overlapping on raw electroencephalogram (EEG) data.

**Figure 2:**
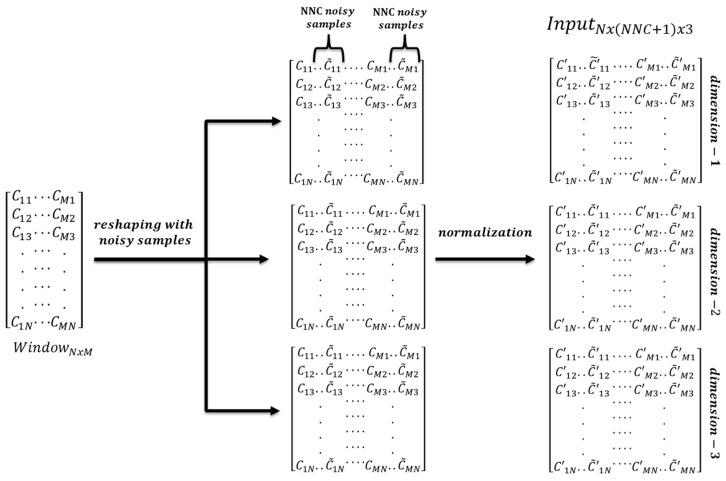
Windowing, Reshaping and Normalization on EEG data.

Data are split into fixed length (N) windows with an overlapping size of N/6 as shown in Figure 1 (displayed for three random channels). One window of EEG data is given in Figure 2 where *M* is the number of selected channels, *C_ab_* is “*b^th^* data point of channel *a*”.

### 4.2. Data Reshaping

EEG data are reshaped to fit the input layer properties of InceptionResnetV2 which is shown in Figure 2. KERAS, which is an open-source neural network library written in Python, is used for the training purpose. Since KERAS is used for training purposes, the minimum input size should be (*N*_1_, *N*, 3) for InceptionResnetV2 where *N*_1_ ≥ 75, *N* ≥ 75 [69]. Depending on the number of selected channels, each channel data are augmented by creating the noisy copies of it. For instance, if the number of selected channels is *S*, for each channel the number of noisy copies is calculated according to Equation (1) where *ceil* operator rounds the number to the next integer (if the number is not integer) and *NNC* is number of noisy copies.

(1) $$NNC=ceil\left(\frac{N1}{S}\right)-1,$$

The noisy copies of each channel are created by adding random samples of a gaussian distribution of mean *μ* and variance *σ*^2^ where *μ* and *σ* are chosen as 0 and 0.01, respectively. This process is given in Equation (2) where $\left[{\tilde{C}}_{a1},\;{\tilde{C}}_{a2},\ldots ,\;{\tilde{C}}_{aN}\right]$ is the noisy copy of the original data $\left[C_{a1},C_{a2},\ldots ,\;C_{aN}\right]$ and $\left[n_{a1},n_{a2},\ldots ,\;n_{aN}\right]$ is the noise vector.

(2) $$\left[{\tilde{C}}_{a1},\;{\tilde{C}}_{a2},\ldots ,\;{\tilde{C}}_{aN}\right]=\left[C_{a1},C_{a2},\ldots ,\;C_{aN}\right]+\left[n_{a1},n_{a2},\ldots ,\;n_{aN}\right].$$

Since the samples are randomly chosen, each noisy copy is different from each other. *N* is related to the windowing size. Therefore, a window size *N* greater than or equal to 75 is chosen. We chose *N* as 300 in order to provide a standard window size for datasets. This corresponds to 1.5 s for the SEED dataset, approximately two seconds for the DEAP dataset and 0.6 s onds for the LUMED dataset. Moreover, we chose *N*_1_ as 80 for all datasets. The augmentation process is repeated three times in order to make the data fit the KERAS input size. This work does not use interpolation between channels because EEG is a nonlinear signal. The reason for adding noise is mainly for data augmentation. There are several ways of data augmentation such as rotation, shifting and adding noise. In the image processing context, rotation, shifting, zooming and adding noise are used. However, we only use noise addition for EEG data augmentation in order to both keep the channel’s original data and create new augmented data with limited gaussian noise. This is as if there was another electrode very close the electrode, which is augmented with additional noise. We use data augmentation instead of data duplication in order to make the network adapt to the noisy data and increase the prediction capability of it. This also prevents the network from overfitting due to data repetition. This technique was similarly applied in [70].

### 4.3. Normalization

Following windowing, augmentation and reshaping, each channel data are normalized by removing mean of each window from each sample. This is repeated for all channels and the noisy copies. The aim of removing the mean is to equate the mean value of each window to 0. This protects the proposed network against probable ill-conditioned situations. In MATLAB, this process is applied automatically on the input data. In KERAS, we performed this manually just before training the network. Each dimension is created separately so they are different from each other.

### 4.4. Channel Selection

In this work, we concentrated on the frontal temporal lobes of the brain. As it is stated in [71,72], emotional changes mostly affect the EEG signals on the frontal and temporal lobes. A different number of channels are tried in this work, and increasing the number of channels does not help improve the accuracy. This is because, technically, including the channels in the model, which are not correlated with the emotion changes, does not help and on the contrary can adversely affect the accuracy. It is also known that the electrical relations between asymmetrical channels are determining the arousal and valence, hence the emotion [73,74]. Therefore, we chose four asymmetrical pairs of electrodes: AF1, F3, F4, F7, T7, AF2, F5, F8 and T8 from frontal and temporal lobes which are equally spread on the skull. The arrangement of these channels in the window is AF1, AF2, F3, F4, F5, F6, F7, T7, T8.

### 4.5. Network Structure

**Figure 3:**
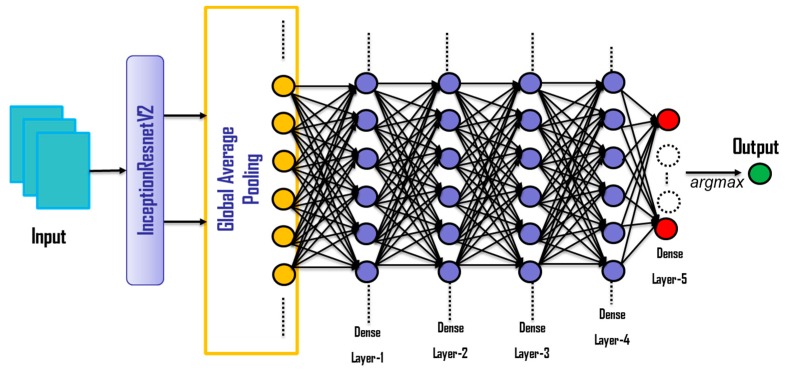
The structure of proposed network model.

In this work a pretrained CNN network, InceptionResnetV2, is used as base model. Following InceptionResnetV2, Global Average Pooling layer is added for decreasing the data dimension and extra dense layers (fully connected layers) are added in order to increase the depth and success for classifying complex data. The overall network structure is given in Figure 3, and the properties of the layers following the CNN is described in Table 1. The training parameters are specified in Table 2. In Figure 3, Dense Layer-5 determines the number of output classes and *argMax* selects the one with the maximum probability. We use the “relu” activation function to cover the interaction effects and non-linearities. This is very important in our problem, while using a deep learning model. Relu is one of the most widely used and successful activation functions in the field of artificial neural networks. Moreover, at the last dense layer, we use the “softmax” activation in order to produce the class probabilities.

### 4.6. Filtering on Output Classes

Since EEG is very prone to noise and different type of artifacts, filtering of EEG signals is widely studied in EEG recognition context. The study conducted in [75] compares three types of smoothing filters (smooth filter, median filter and Savitzky–Golay) on EEG data for the medical diagnostic purposes. The authors concluded that the most useful filter is the classical Savitzky–Golay since it smooths the data without distorting the shape of the waves.

Another EEG data filtering study is provided in [76]. This study employs a moving average filtering on extracted features and then classifies the signal by using an SVM. It achieves very promising accuracy results with limited processing time compared to similar studies.

**Figure 4:**
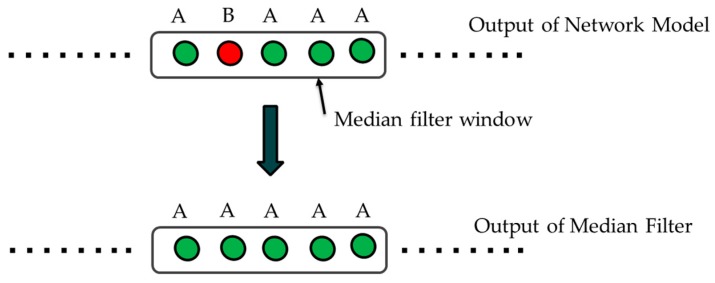
Filtering on Output.

Emotions change quicker than moods for healthy people [77]. However, in very short time intervals (in the range of few seconds), the emotions show lesser variance in healthy individuals with good emotion regulation. Different from the studies [75,76], the filtering is applied on the output in our method. It is assumed that in a defined small-time interval *T* the emotion state does not change. Therefore, we apply a median filter on the output data inside a specific time interval with an aim of removing the false alarms and increase the overall emotion classification accuracy. This process is shown in Figure 4 where A and B stands for different classes.

**Figure 5:**
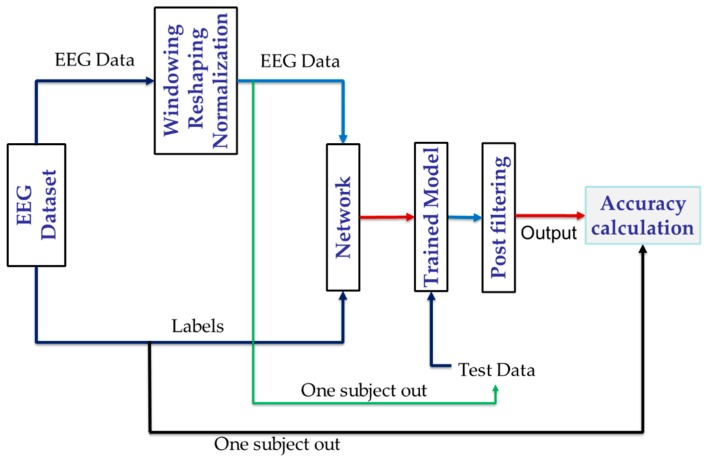
Overall training and testing process of the EEG-based emotion recognition model.

Finally, the overall process that describes how model training and testing is carried out is visually depicted in Figure 5.

## 5. Results and Discussions

**Figure 6:**
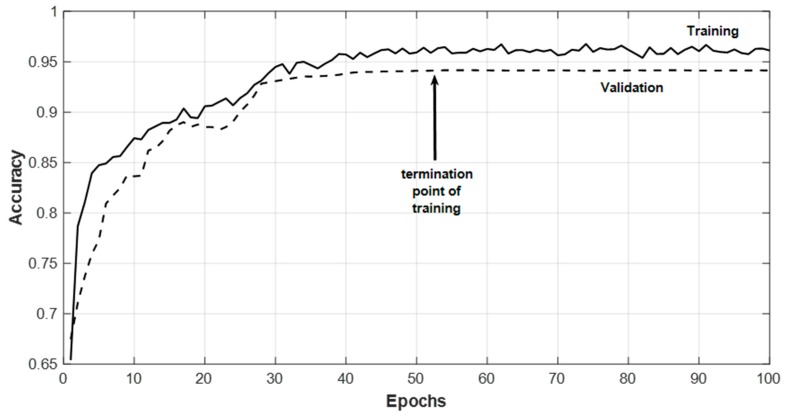
An example of network training.

In this work, for SEED dataset, classification tests are conducted for two categories of classification: two-classes: Positive–Negative valence (Pos–Neg) and three-classes: Positive–Neutral–Negative valence (Pos–Neu–Neg). SEED dataset provides the labels as negative, neutral and positive. DEAP dataset labels the valence and arousal between one and nine. The valence values above 4.5 are taken as positive and the values smaller than 4.5 are taken as negative. For the LUMED dataset, classification is completed as either positive or negative valence. One-subject-out classifications for each dataset are conducted and the results are compared to several reference studies, which provide cross-subject and cross-dataset results. In one-subject-out tests, one subject’s data are excluded completely from the training set. The remaining training set is divided into training and validation sets. In this work, during training, when we do not see improvement on validation accuracy for six consecutive epochs, we stopped the training and applied the test data on the final model. An example is shown in Figure 6. For each user, Table 3 depicts one-subject-out tests for the SEED dataset based on all sessions together, with and without normalization and with and without output filtering. We also obtained the accuracy results without pooling and dense layers. The mean accuracies dropped by 8.3% and 11.1% without pooling and dense layers, respectively. Applying the median filter on the predicted output improves the mean accuracy by approximately 4% for the SEED dataset. The filter size is empirically and set to five. This corresponds approximately to six seconds of data. In this time interval it is assumed that the emotion state remains unchanged. It can be seen in Table 3 that the accuracy for some users is high and for some users it is relatively lower. This is based on the modeling of the network with the remaining training data after excluding the test data. However, standard deviation is still acceptable. Another issue is that when the number of classes is increased from two (Pos–Neg) to three (Pos–Neg–Neu), the prediction accuracies drop. This is because some samples labelled as neutral might fall into the negative or the positive classes.

One of the most important characteristics of our work in this paper is that we provide the accuracy scores for each subject separately. This is not observed in most of the other reference studies that tackle EEG-based emotion recognition.

Table 4 shows the cross-subject accuracy comparison of several top studies, which provides the results for 2-classes (Pos–Neg) or 3-classes (Pos–Neu–Neg). For Pos–Neg, the proposed method achieves 86.5% accuracy which is slightly lower than ST-SBSSVM [48]. However, our method has far less complexity, since it does not depend on pre-feature extraction and associated complex calculations. Furthermore, it is not clear in [48] if the reported maximum accuracy of ST-SBSSVM corresponds to the mean prediction accuracy of all subjects, or the maximum prediction accuracy of any subject amongst all.

Another issue is that many reference cross-subject studies use the excluded users’ data for validation during the training process. In domain adaptation methods, target domain is also used with source domain to convert data into an intermediate common subspace that makes distributions of target and source domain closer. Similar approach with adding an error function is used in [50]. Using the target domain with source domain can increase the cross-subject [50,62] accuracy because of that the distributions between labelled and unlabeled data are controlled by some cost functions empirically. However, these kinds of approaches are not well-directed, as we should know that we only have the source domain in cross-subject and/or cross-dataset classification. We aim to generate a model and feature set only from source domain which will be tested with unused target data (either labelled or unlabeled). Validation and training data should be clearly split, where excluded subjects’ data should not be used in the validation. After reaching the furthest epoch, where overfitting does not kick in yet, training should be stopped. Then, the final trained model should be tested with the excluded subjects’ data. In our study, we respected this rule.

Table 5 shows the accuracy results of proposed model for the DEAP database for two-classes (Pos-Neg). Generally, the reported accuracies are lower than the ones achieved in the SEED dataset. This may be due to the poorer labelling quality of the samples in the DEAP dataset. Some reference studies employ varying re-labelling strategies on the samples of the DEAP dataset to revise class labels. This automatically increases the reported prediction accuracy levels. However, we decided not to alter and respect the original labelling strategy used in that dataset. We only set the threshold in the exact midpoint of the scale of one to nine to divide the samples into two classes, positive and negative. It is acceptable to achieve slightly lower accuracy values than some others as shown in Table 6. To reiterate, post median filtering improves the mean prediction accuracy by approximately 4%.

Table 6 shows the prediction accuracies of several studies that use the DEAP dataset for two classes (Pos-Neg). Our proposed method yields promising accuracy results with only limited complexity (e.g., without any pre-feature extraction cycle) when compared to others. For all eight incoming EEG data channels, the windowing, reshaping, normalization and classification processes take on average 0.34 sec on the test workstation (Core i-9, 3.6 GHz, 64 Gb RAM). This is the computational time used by the Python script and KERAS framework. Hence, the data classification can be achieved with roughly a delay of half a second, rendering our method usable in real-time systems.

Table 7 shows the accuracy results of our proposed model on the LUMED dataset for two-classes (Pos-Neg). It produces a mean prediction accuracy of 81.8% with a standard deviation of 10.9. Post media filtering increases the mean accuracy by approximately 4.5%.

In this work, cross-dataset tests are also conducted between the SEED–DEAP, SEED–LUMED and DEAP–LUMED datasets for positive and negative labels. Table 8 shows the cross-dataset accuracy results between the SEED and DEAP. Our model is trained using the data in the SEED dataset and tested on the DEAP dataset separately. It yields 58.10% mean prediction accuracy, which is promising in this context. The comparison of the cross-dataset performance of our proposed model with the other cross-dataset studies is shown in Table 9. The cross-dataset accuracy of our model is consistently superior to other studies. Table 10 shows the cross-dataset results between SEED–LUMED and DEAP–LUMED. Since LUMED is a new dataset, we cannot give any benchmark results with other studies. However, the mean accuracy results and standard deviations are promising.

## 6. Conclusions

In many recognition and classification problems, the most time and resource consuming aspect is the feature extraction process. Many scientists focus on extracting meaningful features from the EEG signals either in time and/or frequency domains in order to achieve successful classification results. However, the derived feature sets, which can be useful in the classification problem for one subject, recording session or dataset can fail for different subjects, recording sessions and datasets. Furthermore, since the feature extraction process is a complex and time-consuming process, it is not particularly suitable for online and real-time classification problems. In this study, we do not rely on a separate pre-feature extraction process and shift this task to the deep learning cycle that inherently employs this process. Hence, we do not manually remove any potentially useful information from the raw EEG channels. Similar approaches, where deep neural networks are utilized for recognition, were applied in different domains, such as in [83] where electromagnetic sources can be recognized. The success of CNNs has already been shown as highly competent in various classification tasks, especially in the image classification context. Therefore, we deploy of a pretrained CNN architecture called InceptionResnetV2 to classify the EEG data. We have taken the necessary steps to reshape the input data to feed into and train this network.

One of the most important issues, which influences the success of deep learning approaches is the data themselves and the quality and reliability of the labels of the data. The “Brouwer recommendations” about data collection given in [19] are very useful for handling accurate data and labelling. Particularly during the EEG data recording process, these recommendations should be double-checked due to the EEG recording device’s sensitivity to noise.

EEG signals are non-stationary and nonlinear. This makes putting forth a general classification model and a set of features based on the well-studied spectral band powers difficult. It is important to be able to identify stable feature sets between subjects, recording sessions and datasets. Since CNN is very successful at extracting not-so-obvious features from the input data for complex classification problems, we exploit a state-of-the-art pretrained CNN model called InceptionResnetV2, and do not filter out any information from the raw EEG signals. For robustness, we further enrich this deep network by adding fully connected dense layers. This increases the depth and prevents the network from falling into probable ill-conditions and overfitting problems.

In this work, we applied the model successfully on three different EEG datasets: SEED, DEAP and LUMED. Furthermore, we tested our model in a cross-dataset context. We trained our model with the SEED dataset, tested on the DEAP and LUMED datasets. Moreover, we trained our model with the DEAP dataset and tested on the LUMED dataset. We showed that the results are promising and superior to most of the reference techniques. Once we generate the fully pretrained model, we can feed any online raw data directly as inputs to get the output class immediately. Since there is no dedicated pre-feature extraction process, our model is more suitable to be deployed in real-time applications.

## Figures and Tables

**Table 1:** The properties of Layers following InceptionResnetV2 base model.

Layer (Type)	Output Shape	Connected to	Activation Function
Global_Average_Pooling	(None, 1536)	convolution	-
Dense 1	(None, 1024)	Global_Average_Pooling	Relu
Dense 2	(None, 1024)	Dense 1	Relu
Dense 3	(None, 1024)	Dense 2	Relu
Dense 4	(None, 512)	Dense 3	Relu
Dense 5	(None, z ^1^)	Dense 4	Softmax

^1^ z is set according to the number of output classes.

**Table 2:** The training parameters of network.

Property	Value
Base model	InceptionResnetV2
Additional layers	Global Average Pooling, 5 Dense Layers
Regularization	L2
Optimizer	Adam
Loss	Categorical cross entropy
Max. # Epochs	100
Shuffle	True
Batch size	64
Environment	Win 10, 2 Parallel GPU(s), TensorFlow
# Output classes	2 (Pos-Neg) or 3 (Pos-Neu-Neg)

**Table 3:** “One subject out” classification accuracies for the SEED dataset.

Users	Accuracy (Pos-Neg)^1^	Accuracy (without Normalization) (Pos-Neg)	Accuracy (with Filtering) (Pos-Neg)	Accuracy (Pos-Neu-Neg)^2^	Accuracy (without Normalization) (Pos-Neu-Neg)	Accuracy (with Filtering) (Pos-Neu-Neg)
User 1	85.7	74.2	88.5	73.3	55.2	78.2
User 2	83.6	76.3	86.7	72.8	57.4	78.5
User 3	69.2	56.7	74.3	61.6	53.9	67.7
User 4	95.9	69.4	96.1	83.4	72.4	88.3
User 5	78.4	70.1	83.2	74.1	54.3	76.5
User 6	95.8	81.8	96.4	85.3	67.7	89.1
User 7	72.9	56.2	77.7	64.4	53.5	70.3
User 8	69.2	49.3	75.2	62.9	51.3	69.2
User 9	88.6	61.5	90.5	79.2	64.8	82.7
User 10	77.8	70.1	82.7	69.3	56.0	74.5
User 11	78.6	65.7	83.1	73.0	59.9	78.1
User 12	81.6	72.0	85.7	75.6	63.2	78.4
User 13	91.2	80.2	94.2	81.3	73.1	84.9
User 14	86.4	72.3	91.8	73.9	61.1	78.5
User 15	89.2	73.9	92.3	75.4	60.3	80.3
**Average**	**82.94**	**68.64**	**86.56**	**73.7**	**60.27**	**78.34**
**Std.Dev.**	**8.32**	**8.88**	**6.94**	**6.80**	**6.59**	**6.11**

^1^ Pos–Neg: Positive–Negative, ^2^ Pos–Neu–Neg: Positive–Neutral–Negative.

**Table 4:** “One-subject-out” prediction accuracies of reference studies using the SEED dataset.

Work	Accuracy (Pos-Neg)	Accuracy (Pos-Nue-Neg)
ST-SBSSVM [48]	89.0	-
RGNN [55]	-	85.3
Proposed	86.5	78.3
CNN-DDC [50]	-	82.1
ASFM [62]	-	80.4
SAAE [47]	-	77.8
TCA [46]	-	71.6
GFK [78]		67.5
KPCA [79]		62.4
MIDA [80]		72.4

**Table 5:** “One-subject-out” prediction accuracies for DEAP dataset using two-classes (Pos-Neg).

Users	Accuracy	Accuracy (with Median Filtering)
User 1	65.1	69.2
User 2	71.2	73.4
User 3	67.8	69.1
User 4	61.7	65.3
User 5	73.1	75.9
User 6	82.5	85.4
User 7	75.5	77.2
User 8	67.6	71.3
User 9	62.8	67.9
User 10	61.9	66.6
User 11	68.8	72.5
User 12	64.3	69.8
User 13	69.1	74.9
User 14	64.3	68.8
User 15	65.6	70.2
User 16	68.7	72.1
User 17	65.6	70.7
User 18	75.8	78.3
User 19	66.9	72.1
User 20	70.4	73.2
User 21	64.5	68.8
User 22	61.6	68.3
User 23	80.7	83.6
User 24	62.5	69.4
User 25	64.9	70.1
User 26	69.7	72.9
User 27	82.7	85.3
User 28	68.9	73.8
User 29	61.7	69.9
User 30	72.9	77.7
User 31	73.1	78.4
User 32	63.6	68.1
**Average**	**68.60**	**72.81**
**Std. Dev.**	**5.85**	**5.07**

**Table 6:** One-subject-out accuracy comparison of several studies for the DEAP dataset (Pos-Neg).

Work	Accuracy
FAWT [53]	79.9
T-RFE [54]	78.7
Proposed	72.8
ST-SBSSVM [48]	72
VMD-DNN [51]	62.5
MIDA [80]	48.9
TCA [46]	47.2
SA [81]	38.7
ITL [82]	40.5
GFK [78]	46.5
KPCA [79]	39.8

**Table 7:** One-subject-out prediction accuracies for the LUMED dataset.

Users	Accuracy	Accuracy (with Filtering)
User 1	85.8	87.1
User 2	56.3	62.7
User 3	82.2	86.4
User 4	73.8	78.5
User 5	92.1	95.3
User 6	67.8	74.1
User 7	66.3	71.4
User 8	89.7	93.5
User 9	86.3	89.9
User 10	89.1	93.4
User 11	58.9	67.6
**Average**	**77.11**	**81.80**
**Std. Dev.**	**12.40**	**10.92**

**Table 8:** Cross-dataset prediction accuracy results (Trained on SEED and Tested on DEAP).

Users	Accuracy (Pos-Neg)	Accuracy (with Median Filtering) (Pos-Neg)
User 1	50.5	54.9
User 2	61.7	63.7
User 3	43.3	47.3
User 4	46.0	51.5
User 5	68.9	71.9
User 6	45.3	49.4
User 7	73.4	77.2
User 8	51.9	56.3
User 9	62.3	67.9
User 10	63.8	68.6
User 11	48.6	53.6
User 12	46.4	51.3
User 13	50.1	57.1
User 14	70.4	76.9
User 15	58.8	62.8
User 16	59.7	66.3
User 17	46.6	53.1
User 18	64.7	68.5
User 19	47.9	53.3
User 20	39.1	44.6
User 21	62.1	68.8
User 22	45.6	51.3
User 23	61.4	69.9
User 24	54.0	59.2
User 25	50.8	56.3
User 26	40.8	44.7
User 27	39.2	45.3
User 28	42.4	48.4
User 29	46.2	50.3
User 30	41.7	46.2
User 31	61.4	65.7
User 32	53.8	57.1
**Average**	**53.08**	**58.10**
**Std. Dev.**	**9.54**	**9.51**

**Table 9:** One-subject-out cross-dataset prediction accuracy and standard deviation comparison of several studies (Trained on the SEED and tested on the DEAP).

Work	Accuracy (Pos-Neg)	Standard Deviation
Proposed	58.10	9.51
MIDA [80]	47.1	10.60
TCA [46]	42.6	14.69
SA [81]	37.3	7.90
ITL [82]	34.5	13.17
GFK [78]	41.9	11.33
KPCA [79]	35.6	6.97

**Table 10:** Cross-dataset prediction accuracy results (Trained on the SEED/DEAP and tested on the LUMED).

Users (LUMED)	Trained on SEED		Trained on DEAP	
	Accuracy (Pos-Neg)	Accuracy (with Median Filtering) (Pos-Neg)	Accuracy (Pos-Neg)	Accuracy (with Median Filtering) (Pos-Neg)
User 1	68.2	72.3	42.7	48.3
User 2	54.5	61.7	50.4	56.8
User 3	54.3	59.6	49.7	54.1
User 4	59.6	64.1	51.3	57.9
User 5	44.8	53.7	87.1	89.7
User 6	67.1	73.5	52.6	58.4
User 7	53.2	60.8	53.8	59.2
User 8	64.5	71.2	46.0	49.3
User 9	48.6	50.9	84.7	85.6
User 10	64.9	76.3	51.5	58.8
User 11	57.1	64.8	63.8	67.1
**Average**	**57.89**	**64.44**	**57.6**	**62.29**
**Std. Dev.**	**7.33**	**7.82**	**14.23**	**12.91**

## References

[1] Top 14 EEG Hardware Companies. https://imotions.com/blog/top-14-eeg-hardware-companies-ranked/.

[2] Jurcak V., Tsuzuki D., Dan I. (2007). 10/20, 10/10, and 10/5 systems revisited: Their validity as relative head-surface-based positioning systems. NeuroImage.

[3] Aboalayon K.A.I., Faezipour M., Almuhammadi W.S., Moslehpour S. (2016). Sleep Stage Classification Using EEG Signal Analysis: A Comprehensive Survey and New Investigation. Entropy.

[4] Acharya U., Sree S.V., Swapna G., Martis R.J., Suri J.S. (2013). Automated EEG analysis of epilepsy: A review. Knowl. Based Syst..

[5] Engemann D., Raimondo F., King J.-R., Rohaut B., Louppe G., Faugeras F., Annen J., Cassol H., Gosseries O., Slezak D.F. (2018). Robust EEG-based cross-site and cross-protocol classification of states of consciousness. Brain.

[6] Arns M., Conners C.K., Kraemer H.C. (2013). A decade of EEG theta/beta ratio research in ADHD: A meta-analysis. J. Atten. Disord..

[7] Liu N.-H., Chiang C.-Y., Chu H.-C. (2013). Recognizing the Degree of Human Attention Using EEG Signals from Mobile Sensors. Sensors.

[8] Shestyuk A., Kasinathan K., Karapoondinott V., Knight R.T., Gurumoorthy R. (2019). Individual EEG measures of attention, memory, and motivation predict population level TV viewership and Twitter engagement. PLoS ONE.

[9] Mohammadpour M., Mozaffari S. Classification of EEG-based attention for brain computer interface. Proceedings of the 3rd Iranian Conference on Intelligent Systems and Signal Processing (ICSPIS).

[10] So W.K.Y., Wong S., Mak J.N., Chan R.H.M. (2017). An evaluation of mental workload with frontal EEG. PLoS ONE.

[11] Thejaswini S., Ravikumar K.M., Jhenkar L., Aditya N., Abhay K.K. (2019). Analysis of EEG Based Emotion Detection of DEAP and SEED-IV Databases Using SVM. Int. J. Recent Technol. Eng..

[12] Liu J., Meng H., Nandi A., Li M. Emotion detection from EEG recordings. Proceedings of the 12th International Conference on Natural Computation, Fuzzy Systems and Knowledge Discovery (ICNC-FSKD).

[13] Gómez A., Quintero L., López N., Castro J. (2016). An approach to emotion recognition in single-channel EEG signals: A mother child interaction. J. Phys. Conf. Ser..

[14] Xing X., Li Z., Xu T., Shu L., Hu B., Xu X. (2019). SAE+LSTM: A New Framework for Emotion Recognition From Multi-Channel EEG. Front. Neurorobot..

[15] Müller-Putz G., Peicha L., Ofner P. Movement Decoding from EEG: Target or Direction. Proceedings of the 7th Graz Brain-Computer Interface Conference.

[16] Padfield N., Zabalza J., Zhao H., Masero V., Ren J. (2019). EEG-Based Brain-Computer Interfaces Using Motor-Imagery: Techniques and Challenges. Sensors.

[17] Mondini V., Mangia A.L., Cappello A. (2016). EEG-Based BCI System Using Adaptive Features Extraction and Classification Procedures. Comput. Intell. Neurosci..

[18] Picard R.W. (1995). Affective Computing.

[19] Alarcao S.M., Fonseca M.J. (2019). Emotions Recognition Using EEG Signals: A Survey. IEEE Trans. Affect. Comput..

[20] Ekman P., Friesen W.V., O’Sullivan M., Chan A., Diacoyanni-Tarlatzis I., Heider K., Krause R., Lecompte W.A., Pitcairn T., Ricci-Bitti P.E. (1987). Universals and cultural differences in the judgments of facial expressions of emotion. J. Pers. Soc. Psychol..

[21] Scherer K.R. (2005). What are emotions? And how can they be measured?. Soc. Sci. Inf..

[22] Koelstra S., Muhl C., Soleymani M., Lee J.-S., Yazdani A., Ebrahimi T., Pun T., Nijholt A., Patras I. (2011). DEAP: A Database for Emotion Analysis; Using Physiological Signals. IEEE Trans. Affect. Comput..

[23] Russell J.A. (1980). A circumplex model of affect. J. Pers. Soc. Psychol..

[24] Aydin S.G., Kaya T., Guler H. (2016). Wavelet-based study of valence-arousal model of emotions on EEG signals with LabVIEW. Brain Inform..

[25] Paltoglou G., Thelwall M. (2012). Seeing Stars of Valence and Arousal in Blog Posts. IEEE Trans. Affect. Comput..

[26] The McGill Physiology Virtual Lab. https://www.medicine.mcgill.ca/physio/vlab/biomed_signals/eeg_n.htm.

[27] Al-Fahoum A.S., Al-Fraihat A.A. (2014). Methods of EEG Signal Features Extraction Using Linear Analysis in Frequency and Time-Frequency Domains. ISRN Neurosci..

[28] Duan R.-N., Zhu J.-Y., Lu B.-L. Differential entropy feature for EEG-based emotion classification. Proceedings of the 6th International IEEE/EMBS Conference on Neural Engineering (NER).

[29] Zheng W.-L., Lu B.-L. (2015). Investigating Critical Frequency Bands and Channels for EEG-Based Emotion Recognition with Deep Neural Networks. IEEE Trans. Auton. Ment. Dev..

[30] George F.P., Shaikat I.M., Hossain P.S.F., Parvez M.Z., Uddin J. (2019). Recognition of emotional states using EEG signals based on time-frequency analysis and SVM classifier. Int. J. Electr. Comput. Eng..

[31] Soundarya S. (2019). An EEG based emotion recognition and classification using machine learning techniques, I. J. Emerg. Technol. Innov. Eng..

[32] Swati V., Preeti S., Chamandeep K. (2015). Classification of Human Emotions using Multiwavelet Transform based Features and Random Forest Technique. Indian J. Sci. Technol..

[33] Bono V., Biswas D., Das S., Maharatna K. Classifying human emotional states using wireless EEG based ERP and functional connectivity measures. Proceedings of the IEEE-EMBS International Conference on Biomedical and Health Informatics (BHI).

[34] Nattapong T., Ken-ichi F., Masayuki N. Application of Deep Belief Networks in EEG, based Dynamic Music-emotion Recognition. Proceedings of the 2016 International Joint Conference on Neural Networks (IJCNN).

[35] Prieto L.A.B., Oplatková Z.K. (2018). Emotion Recognition using AutoEncoders and Convolutional Neural Networks. MENDEL.

[36] Chen J.X., Zhang P.W., Mao Z.J., Huang Y.F., Jiang D.M., Zhang Y.N. (2019). Accurate EEG-Based Emotion Recognition on Combined Features Using Deep Convolutional Neural Networks. IEEE Access.

[37] Tripathi S., Acharya S., Sharma R.D., Mittal S., Bhattacharya S. Using deep and convolutional neural networks for accurate emotion classification on DEAP Dataset. Proceedings of the Thirty-First AAAI Conference on Artificial Intelligence.

[38] Salama E.S., El-Khoribi R.A., Shoman M.E., Shalaby M.A. (2018). EEG-based emotion recognition using 3D convolutional neural networks. Int. J. Adv. Comput. Sci. Appl..

[39] Alhagry S., Fahmy A.A., El-Khoribi R.A. (2017). Emotion Recognition based on EEG using LSTM Recurrent Neural Network. Int. J. Adv. Comput. Sci. Appl..

[40] Jeevan R.K., Kumar P.S., Srivikas M., Rao S.V.M. EEG-based emotion recognition using LSTM-RNN machine learning algorithm. Proceedings of the 1st International Conference on Innovations in Information and Communication Technology (ICIICT).

[41] Li M., Xu H., Liu X., Lu S. (2018). Emotion recognition from multichannel EEG signals using K-nearest neighbor classification. Technol. Health Care.

[42] DEAP Dataset. https://www.eecs.qmul.ac.uk/mmv/datasets/deap/.

[43] Alazrai R., Homoud R., Alwanni H., Daoud M.I. (2018). EEG-Based Emotion Recognition Using Quadratic Time-Frequency Distribution. Sensors.

[44] Nivedha R., Brinda M., Vasanth D., Anvitha M., Suma K.V. EEG based emotion recognition using SVM and PSO. Proceedings of the International Conference on Intelligent Computing, Instrumentation and Control Technologies (ICICICT).

[45] Particle Swarm Optimization. https://www.sciencedirect.com/topics/engineering/particle-swarm-optimization.

[46] Pan S.J., Tsang I.W., Kwok J.T., Yang Q. (2010). Domain Adaptation via Transfer Component Analysis. IEEE Trans. Neural Netw..

[47] Chai X., Wang Q., Zhao Y., Liu X., Bai O., Li Y. (2016). Unsupervised domain adaptation techniques based on auto-encoder for non-stationary EEG-based emotion recognition. Comput. Boil. Med..

[48] Yang F., Zhao X., Jiang W., Gao P., Liu G. (2019). Multi-method Fusion of Cross-Subject Emotion Recognition Based on High-Dimensional EEG Features. Front. Comput. Neurosci..

[49] SEED Dataset. http://bcmi.sjtu.edu.cn/~seed/.

[50] Zhang W., Wang F., Jiang Y., Xu Z., Wu S., Zhang Y. (2019). Cross-Subject EEG-Based Emotion Recognition with Deep Domain Confusion. ICIRA 2019 Intell. Robot. Appl..

[51] Pandey P., Seeja K. (2019). Subject independent emotion recognition from EEG using VMD and deep learning. J. King Saud Univ. Comput. Inf. Sci..

[52] Keelawat P., Thammasan N., Kijsirikul B., Numao M. Subject-Independent Emotion Recognition During Music Listening Based on EEG Using Deep Convolutional Neural Networks. Proceedings of the IEEE 15th International Colloquium on Signal Processing & Its Applications (CSPA).

[53] Gupta V., Chopda M.D., Pachori R.B. (2018). Cross-Subject Emotion Recognition Using Flexible Analytic Wavelet Transform From EEG Signals. IEEE Sens. J..

[54] Yin Z., Wang Y., Liu L., Zhang W., Zhang J. (2017). Cross-Subject EEG Feature Selection for Emotion Recognition Using Transfer Recursive Feature Elimination. Front. Neurorobot..

[55] Zhong P., Wang D., Miao C. (2019). EEG-Based Emotion Recognition Using Regularized Graph Neural Networks. arXiv.

[56] Pan S.J., Yang Q. (2009). A Survey on Transfer Learning. IEEE Trans. Knowl. Data Eng..

[57] Krauledat M., Tangermann M., Blankertz B., Müller K.-R. (2008). Towards Zero Training for Brain-Computer Interfacing. PLoS ONE.

[58] Fazli S., Popescu F., Danóczy M., Blankertz B., Müller K.-R., Grozea C. (2009). Subject-independent mental state classification in single trials. Neural Netw..

[59] Kang H., Nam Y., Choi S. (2009). Composite Common Spatial Pattern for Subject-to-Subject Transfer. IEEE Signal Process. Lett..

[60] Zheng W.-L., Zhang Y.-Q., Zhu J.-Y., Lu B.-L. Transfer components between subjects for EEG-based emotion recognition. Proceedings of the 2015 International Conference on Affective Computing and Intelligent Interaction (ACII).

[61] Zheng W.-L., Lu B.-L. Personalizing EEG-based affective models with transfer learning. Proceedings of the Twenty-Fifth International Joint Conference on Artificial Intelligence.

[62] Chai X., Wang Q., Zhao Y., Li Y., Wang Q., Liu X., Bai O. (2017). A Fast, Efficient Domain Adaptation Technique for Cross-Domain Electroencephalography (EEG)-Based Emotion Recognition. Sensors.

[63] Lan Z., Sourina O., Wang L., Scherer R., Müller-Putz G.R. (2019). Domain Adaptation Techniques for EEG-Based Emotion Recognition: A Comparative Study on Two Public Datasets. IEEE Trans. Cogn. Dev. Syst..

[64] Tzeng E., Hoffman J., Zhang N., Saenko K., Darrell T. (2014). Deep domain confusion: Maximizing for domain invariance. arXiv.

[65] Loughborough University EEG based Emotion Recognition Dataset. https://www.dropbox.com/s/xlh2orv6mgweehq/LUMED_EEG.zip?dl=0.

[66] Plöchl M., Ossandón J.P., König P. (2012). Combining EEG and eye tracking: Identification, characterization, and correction of eye movement artifacts in electroencephalographic data. Front. Hum. Neurosci..

[67] Enobio 8. https://www.neuroelectrics.com/solutions/enobio/8/.

[68] Pretrained Deep Neural Networks. https://uk.mathworks.com/help/deeplearning/ug/pretrained-convolutional-neural-networks.html.

[69] Keras Applications. https://keras.io/applications/#inceptionresnetv2.

[70] Wang F., Zhong S.-H., Peng J., Jiang J., Liu Y., Schoeffmann K., Chalidabhongse T.H., Ngo C.W., Aramvith S., O’Connor N.E., Ho Y.-S., Gabbouj M., Elgamma A. (2018). Data Augmentation for EEG-Based Emotion Recognition with Deep Convolutional Neural Networks. MultiMedia Modeling, Proceedings of the 24th International Conference, MMM 2018, Bangkok, Thailand, 5–7 February 2018.

[71] Salzman C.D., Fusi S. (2010). Emotion, cognition, and mental state representation in amygdala and prefrontal cortex. Annu. Rev. Neurosci..

[72] Zhao G., Zhang Y., Ge Y. (2018). Frontal EEG Asymmetry and Middle Line Power Difference in Discrete Emotions. Front. Behav. Neurosci..

[73] Bos D.O. (2006). EEG-based Emotion Recognition: The influence of Visual and Auditory Stimuli. http://citeseerx.ist.psu.edu/viewdoc/download?doi=10.1.1.226.8188&rep=rep1&type=pdf.

[74] Alnafjan A., Hosny M., Al-Wabil A., Alohali Y.A. (2017). Classification of Human Emotions from Electroencephalogram (EEG) Signal using Deep Neural Network. Int. J. Adv. Comput. Sci. Appl..

[75] Kawala-Sterniuk A., Podpora M., Pelc M., Blaszczyszyn M., Gorzelanczyk E.J., Martinek R., Ozana S. (2020). Comparison of Smoothing Filters in Analysis of EEG Data for the Medical Diagnostics Purposes. Sensors.

[76] Tang C., Wang D., Tan A.-H., Miao C. EEG-Based Emotion Recognition via Fast and Robust Feature Smoothing. Proceedings of the 2017 International Conference on Brain Informatics.

[77] Beedie C., Terry P., Lane A. (2005). Distinctions between emotion and mood. Cogn. Emot..

[78] Gong B., Shi Y., Sha F., Grauman K. Geodesic flow kernel for unsupervised domain adaptation. Proceedings of the 2012 IEEE Conference on Computer Vision and Pattern Recognition.

[79] Schölkopf B., Smola A., Müller K.-R. (1998). Nonlinear Component Analysis as a Kernel Eigenvalue Problem. Neural Comput..

[80] Yan K., Kou L., Zhang D. (2018). Learning Domain-Invariant Subspace Using Domain Features and Independence Maximization. IEEE Trans. Cybern..

[81] Fernando B., Habrard A., Sebban M., Tuytelaars T. Unsupervised Visual Domain Adaptation Using Subspace Alignment. Proceedings of the 2013 IEEE International Conference on Computer Vision.

[82] Shi Y., Sha F. Information-Theoretical Learning of Discriminative Clusters for Unsupervised Domain Adaptation. Proceedings of the 2012 International Conference on Machine Learning (ICML).

[83] Matuszewski J., Pietrow D. Recognition of electromagnetic sources with the use of deep neural networks. Proceedings of the XII Conference on Reconnaissance and Electronic Warfare Systems.

